# Increased functional network segregation in glioma patients posttherapy: A neurological compensatory response or catastrophe for cognition?

**DOI:** 10.1162/netn_a_00449

**Published:** 2025-06-27

**Authors:** Laurien De Roeck, Rob Colaes, Patrick Dupont, Stefan Sunaert, Steven De Vleeschouwer, Paul M. Clement, Charlotte Sleurs, Maarten Lambrecht

**Affiliations:** Department of Radiation-Oncology, University Hospitals Leuven, Belgium; Department of Oncology, Katholieke Universiteit Leuven, Belgium; Department of Imaging & Pathology, Katholieke Universiteit Leuven, Belgium; Leuven Brain Institute, Katholieke Universiteit Leuven, Belgium; Department of Neurosciences, Katholieke Universiteit Leuven, Belgium; Department of Neurosurgery, University Hospitals Leuven, Belgium; Department of General Medical Oncology, University Hospitals Leuven, Belgium; Department of Cognitive Neuropsychology, Tilburg University, The Netherlands

**Keywords:** Glioma, Cognition, Graph theory, Hubs, Resting-state functional MRI, Functional connectivity

## Abstract

The brain operates through networks of interconnected regions, which can be disrupted by glial tumors and their treatment. This study investigates associations between this altered functional network topology and cognition in gliomas. We studied 50 adult glioma survivors (>1-year posttherapy) and 50 healthy controls. Participants underwent cognitive assessments across six domains and an 8-min resting-state functional MRI. Based on the BOLD signal, partial correlations were computed among 78 brain regions. From their absolute values, whole-brain and nodal graph metrics were derived and normalized to random graphs. Group differences in whole-brain and nodal graph metrics were assessed with Mann–Whitney *U* tests and mixed-design analyses of variance, respectively. Metrics exhibiting significant intergroup differences were correlated with cognitive scores, with *p*_bonf_ < 0.050 indicating significance. Among controls, 8 of 78 nodes were identified as hubs. Patients exhibited significantly higher whole-brain clustering, correlating with intelligence (*r*(98) = −0.409, *p*_bonf_ < 0.001) and executive functioning (*r*(98) = 0.300, *p*_bonf_ = 0.014). Lower centrality, higher nodal clustering, and assortativity were also observed in patients, particularly in hubs, correlating with language and executive functioning, respectively (all *r*(98) > 0.300, *p*_bonf_ < 0.050). Glioma patients commonly experience cognitive deficits alongside posttreatment alterations in functional network topology. Alterations in clustering, assortativity, and centrality may specifically act as compensatory mechanisms, significantly influencing cognitive functioning.

## Author Summary

Cross-disease research has highlighted the importance of functional networks in cognitive functioning. Given that these networks can be disrupted by glial tumors and their treatment, our study examines the relationship between cognitive performance and altered functional network topology using resting-state fMRI in a sizable prospective cohort of 50 glioma survivors and 50 matched healthy controls. With this study, we reveal significant alterations in functional network topology and its hubs in glioma survivors, of which specific graph measures appeared to be specifically valuable in predicting executive functioning and language outcomes. These insights underscore the importance of network-based approaches in future research to better understand and address these deficits, certainly given that functional networks and their hubs are understudied in this population.

## INTRODUCTION

Cognitive sequelae are common, yet poorly understood the long-term side effects of glioma treatment (Armstrong et al., 2016). These symptoms can affect various cognitive domains, including language, memory, attention, and executive functioning. Although associations have been identified between cognitive performance and specific brain structures (Eekers et al., 2018, 2021; Habets et al., 2019; Mitchell et al., 2014; Peiffer et al., 2013), there is growing evidence that higher order cognitive tasks, such as executive functioning, may rather rely on diverse functional brain networks and their interactions (Bosma et al., 2008, 2009; Farahani et al., 2019; Kocher et al., 2020; Maas & Douw, 2023).

Such functional brain networks can be modeled based on functional MRI (fMRI), electroencephalography (EEG), and magnetoencephalography (MEG) (Maas & Douw, 2023), by, for instance, correlating different regional time series. Resting-state fMRI (rsfMRI) has revolutionized our understanding of functional brain networks by allowing to study the brain’s activity noninvasively while not being engaged in any specific task. This technique measures spontaneous fluctuations in blood-oxygen-level-dependent (BOLD) signals, which reflect neuronal activity. By examining these fluctuations, coherent patterns of activity across different brain regions can be identified, often referred to as functional connectivity (Lang et al., 2014).

Brain networks of healthy individuals exhibit a small-world architecture, which is characterized by short average path lengths and high clustering coefficient values, reflecting their optimal structure for information transfer (Watts & Strogatz, 2006). The clustering coefficient evaluates how closely connected a group of nodes or brain region is within a network, reflecting the network’s level of segregation. On the other hand, the average path length measures integration, indicating how easily information can travel across the entire network (Rubinov & Sporns, 2010).

Glial tumors and their treatments can potentially compromise both the local integrity of the brain tissue as well as its functional connectivity (Fox & King, 2018; Volz et al., 2018). Previous studies using rsfMRI have shown that glioma patients exhibit altered functional network topology, characterized by abnormally high clustering between regions, reduced global integration and efficiency, and disintegration of the default mode network in both the ipsilesional and contralesional hemispheres (De Baene et al., 2017; Derks et al., 2021; Esposito et al., 2012; Harris et al., 2014; Maesawa et al., 2015). While these functional changes such as higher clustering have been associated with better cognitive performance in healthy brain networks (Rubinov & Sporns, 2010; van den Heuvel et al., 2009), lower global integration and higher segregation have also been associated with poorer cognitive performance in networks of glioma patients (Bosma et al., 2009; De Baene et al., 2019; Maesawa et al., 2015; van Lingen et al., 2023).

In addition to these functional network changes, specific brain regions within these networks could be of importance in cognitive outcomes. Since local therapies, such as cranial radiotherapy, can be modified, sparing these regions in treatment plans, these brain structures (or organs) at risk are of particular interest to clinicians.

Functional brain network hubs have been identified as potential cognitive brain structures (Buckner et al., 2009; Crossley et al., 2014; Fagerholm et al., 2015; Jones et al., 2016). This is not surprising, since functional brain networks hubs exhibit an overall higher level of connectivity to most other brain regions (van den Heuvel & Sporns, 2013). Moreover, these hubs play a crucial role in facilitating information transfer and processing, partly due to their propensity to form long-range connections (Arnatkevičiūtė et al., 2018; Harriger et al., 2012; van den Heuvel et al., 2012). Additionally, these hubs are typically located in the association cortices, basal ganglia, and thalamus—regions integral to higher order cognition (Buckner & Krienen, 2013; van den Heuvel & Sporns, 2013).

In this study, we explored the functional network topology and its potential relationship with cognitive functioning in glioma patients and healthy controls. Specifically, we focused on the role of functional hubs in cognitive functioning. Therefore, we examined the association between whole-brain and hub-specific nodal graph metrics and cognitive functioning. By conducting a comprehensive examination of brain network characteristics, we aim to improve the ability to predict the cognitive challenges that brain tumor patients may face in the future.

## MATERIALS AND METHODS

### Subjects

Adult patients proficient in Dutch (aged ≥18 years) who had been treated for a World Health Organization (WHO) Grade 2 or Grade 3 glioma between 2007 and 2019 at the University Hospitals of Leuven (Belgium) were assessed. Eligible participants had a WHO performance status of 0–2, were at least 1 year after surgery and/or chemoradiotherapy, and were undergoing routine clinical follow-up. Excluded from the study were patients with prior diagnoses of other brain neoplasms or psychiatric conditions, intellectual disability, or relapse following chemo- and/or radiation therapy. Healthy controls were sourced from online forums and matched individually with patients based on sex, age (with a maximal age difference of 3 years), and educational level (seven Verhage categories; Verhage, 1964).

### Study Design

All participants underwent a comprehensive assessment involving self-report inventories (30 min), cognitive testing (1 hr), and a neuroimaging protocol (1 hr), administered on the same day. All subjects were scanned between 2021 and 2022. Ethical approval for the study (approval number: S63580) was obtained from the Ethical Committee of the University Hospitals of Leuven. Written informed consent was obtained from all participants included in the study, which was performed according to the Declaration of Helsinki.

### Neuropsychological Assessment

Cognitive functions, covering domains of language (measured by the Controlled Oral Word Association Test; Ruff et al., 1996), memory (assessed using the Hopkins Verbal Learning Test–Revised; Benedict et al., 1998), motor skills (evaluated via the Grooved Pegboard Test; Bryden & Roy, 2005), and attention and executive functioning (examined through the Trail Making Test Parts A & B; Bowie & Harvey, 2006); subtests of the digit span and digit symbol substitution tests of the Wechsler Adult Intelligence Scale, Fourth Edition (WAIS-IV) (Lichtenberger & Kaufman, 2006); and the Stroop Color and Word Test (Scarpina & Tagini, 2017) were assessed. The matrix reasoning subtest of the WAIS-IV was used as a proxy measure of general cognitive ability. While not a direct measure of premorbid intelligence, its nonverbal nature and correlation with overall IQ provide a reasonable approximation (proxy IQ). Raw cognitive test scores were transformed into w-scores, which were adjusted for education and age of the healthy control group (see the Supporting Information for the detailed description; Rijnen et al., 2020). These w-scores were then averaged into these six cognitive domains based on between-test correlations and according to the Diagnostic and Statistical Manual of Mental Disorders, Fifth Edition definitions (Supporting Information Table S1; Sachdev et al., 2014). Cognitive impairment was defined based on the International Cancer and Cognition Task Force recommendations, with two or more w-scores at or below −1.5 or at least one test with a w-score at or below −2.0 (Wefel et al., 2011).

### Image Acquisition

Magnetic resonance (MR) images were acquired on a 3T Philips Achieva scanner equipped with a 32-array head coil. The imaging protocol included T1-weighted images (MPRAGE, Magnetization Prepared RApid Gradient-Echo), 3D FLAIR images (Fluid-attenuated inversion recovery images), and rsfMRI, among other MRI sequences.

Initially, T1-weighted images (MPRAGE) were obtained during a 7-min scan: voxel size = 0.8 × 0.8 × 0.8 mm isotropically, flip angle (FA) = 8°, repetition time (TR) of 5,800 ms, echo time (TE) of 2,500 ms, field of view (FOV) of 320 × 320 voxels, and 208 slices.

Subsequently, a 3D FLAIR scan was performed for 4 min: voxel size = 1.0 × 1.0 × 1.0 mm isotropically, inversion time = 165 ms, FA = 90°, TR/TE = 4,800/340 ms, number of signal averages = 2, FOV = 256 × 256 voxels, and 183 slices.

Finally, rsfMRIs were acquired during a 8-min session, during which participants were instructed to keep still with their eyes closed, refrain from engaging in specific thoughts, and relax without falling asleep (EPI, voxel size = 2.5 mm^3^ isotropically, FA = 70°, TR/TE = 1,000/33 ms, FOV = 240 × 240 × 146 mm, reconstruction matrix = 96 × 96 × 56, 450 volumes, multiband acceleration factor of 4).

### Image Preprocessing

Image quality was assessed using MRI Quality Control tool (MRIQC) by evaluating three specific parameters as described in the work of Ran et al. (2020). Specifically, we assessed framewise displacement with a cutoff value of <0.5 mm, DVARS with a threshold set to the mean of the DVARS values over time plus three standard deviations, and overall translation with limits of <1.5 mm and <1.5° in all directions. The volumes that exceeded these predefined limits underwent scrubbing. If too many volumes required scrubbing, the data were deemed to be of insufficient quality.

**Figure 1.**
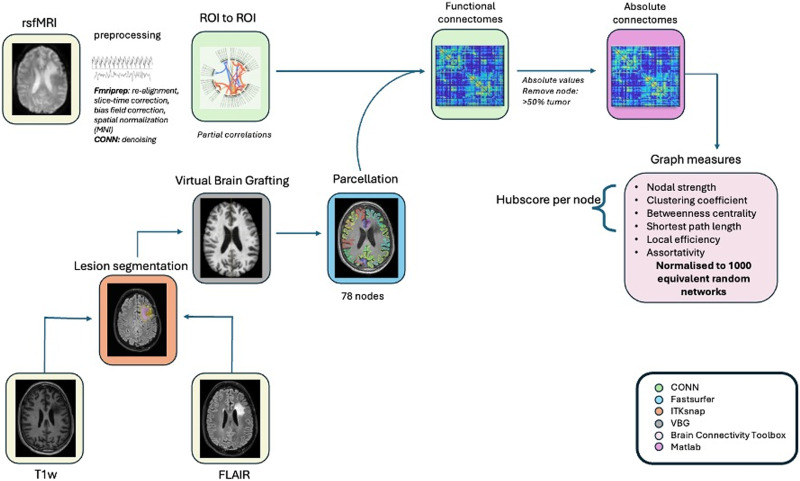
Workflow of imaging processing and analysis.

Image processing was performed using MATLAB-based scripts (MATLAB R2023a and Bash) and validated toolboxes. An overview of the imaging preprocessing and analysis is depicted in Figure 1.

First, tumor lesions were segmented semiautomatically by using resseg (resection cavity; Pérez-García et al., 2021), HD-glio-auto (gliosis and/or residual tumoral tissue; Kickingereder et al., 2019), and FastSurfer (ventricles; Henschel et al., 2020), followed by a manual correction on the FLAIR and T1-weighted images in ITKsnap (v3.6.0; Yushkevich et al., 2019). Virtual Brain Grafting (VBG v0.61; Radwan et al., 2021) was used to create lesion-free and T1-weighted images and masks, which facilitated the parcellation of subcortical (thalamus, caudate, putamen, pallidum, amygdala, hippocampus, and accumbens area) and cortical regions according to the Desikan-Killiany-Tourville (DKT) atlas (Desikan et al., 2006; Klein & Tourville, 2012) using FastSurfer (v2.0; Henschel et al., 2020). This process resulted in 78 parcellations (i.e., network nodes).

Next, functional MR images were visually checked for BOLD signal losses in the frontal and temporal brain regions and were preprocessed using fMRIPrep 23.0 (Esteban et al., 2019), including realignment, slice-time correction, field unwarping, bias field correction, and spatial normalization to the ICBM152 2009c nonlinear asymmetrical template. Subsequently, data were further processed using the CONN toolbox (CONN 20b; Whitfield-Gabrieli & Nieto-Castanon, 2012). This included functional outlier detection (based on scrubbing of motion-affected functional volumes). We addressed potential sources of spurious variance by regressing out the realignment and scrubbing parameters, as well as signals from the white matter and ventricular system. To further minimize the impact of the low-frequency drift and high-frequency physiological noise, we applied linear detrending and temporal band-pass filtering (0.009–0.8 Hz).

### Functional Network Construction

To obtain the representative time series for each region of interest (ROI), individual time series were averaged over the voxels within each parcel. A functional connectivity matrix was then created for each patient by calculating the partial correlations between the time series of each pair of relevant ROIs or network nodes (*n* = 78 × 77; 3,003 edges), using the time series of other regions as control variables. Next, we took the absolute values of these correlations, as previous research has shown them to be more reproducible (Ran et al., 2020), and both negative and positive values contain important biological information (Kazeminejad & Sotero, 2020).

Self-connections were removed from the subject-specific connectomes. Finally, to remove the influence of the compromised connections to the tumor-bearing areas, the connectivity values between >50% tumor-affected regions and unaffected regions were set to 0.

### Graph Measures

Whole-brain weighted graph measures of local efficiency, global efficiency, average path length, and clustering coefficient, together with nodal weighted graph measures of the shortest path length, nodal strength, local efficiency, assortativity, clustering coefficient, and betweenness centrality, were calculated by using an in-house-developed MATLAB script and the Brain Connectivity Toolbox (v2019-03-03; Rubinov & Sporns, 2010).

We chose to include these specific graph measures because betweenness centrality, clustering coefficient, nodal strength, and shortest path length are used to define hubs by calculating a hubscore (van den Heuvel et al., 2010). Additionally, we calculated local efficiency and assortativity due to their previous associations with cognition in neuro-oncological research (De Baene et al., 2019; Sleurs et al., 2021).

In summary, the average path length measures the mean shortest path between all pairs of nodes in a network, providing insights into the overall efficiency of information propagation. The shortest path of a node is the path with minimal cost and the average of the shortest paths from that node to any other node in the network. It was calculated using Dijkstra’s algorithm (Dijkstra, 1959).

The clustering coefficient assesses the degree of local connectivity, indicating how well a node’s neighbors are connected to each other. The global clustering coefficient is the average of all nodes’ clustering coefficients, calculated using the method for weighted networks proposed by Wang et al. (2017).

Local efficiency evaluates the efficiency of information exchange among a node’s immediate neighbors. High local efficiency signifies that the neighbors are well-connected and can efficiently share information. This metric was also calculated according to Wang et al.’s (2017) recommendations. Global efficiency measures the overall interconnectedness of the network.

Nodal strength represents the total influence or importance of a node, based on the strength of its connections. Nodes with higher nodal strength are more central and influential within the network due to their strong interactions with other nodes.

Betweenness centrality measures the frequency of a node appearing on the shortest paths between other nodes, highlighting those crucial for information flow. Assortativity evaluates the tendency of nodes to connect with other nodes that have similar or dissimilar properties.

To account for the influence of weight distribution on graph measures, we normalized the nodal and whole-brain graph metrics for each subject using 1,000 equivalent random graphs that consisted of the same number of nodes and distribution of connectivity values. We then scaled the observed graph measures by dividing them by the corresponding median values derived from these random networks. This approach was chosen because the median, unlike the mean, is not affected by outliers, providing a more robust normalization. The code is available on the Open Science Framework (link).

### Defining Hubs and Nonhubs

Based on the definition of van den Heuvel et al. (2010), an overall hubscore for each node was calculated based on four nodal metrics: betweenness centrality, shortest path length, clustering coefficient, and nodal strength. Nodes that ranked in the top 20% for betweenness centrality and nodal strength, and the bottom 20% for shortest path length and clustering coefficient, were graded with one point for each criterion met, resulting in a hubscore ranging from 0 to 4. Nodes achieving a total score of 2 or more in over 50% of healthy controls were classified as network hubs (van den Heuvel et al., 2010). A nonhub was defined as a node with a hubscore of 0 in over 50% of healthy controls.

### Statistical Analysis

To investigate differences in whole-brain graph metrics (local efficiency, global efficiency, characteristic path length, and clustering coefficient) between groups, we utilized nonparametric Mann–Whitney *U* tests. To assess the disparity in the frequency of hub regions between groups, chi-square tests were performed.

We then examined nodal graph metrics, comparing patients and controls, for hub and nonhub nodes separately, using two mixed-design analyses of variance (ANOVAs). Log transformations were applied to nonnormally distributed graph measures prior to analysis. For cases where the assumption of sphericity was violated, the Greenhouse–Geisser correction was used.

Subsequently, we explored the associations between log-transformed graph measures and cognitive outcomes. In a post hoc analysis, we repeated the analysis with age as a covariate. Graph measures that significantly differed between groups, in both hubs and nonhubs, were correlated with w-scores across six cognitive domains, using Pearson correlation coefficients across the whole sample. Additionally, correlation matrices were calculated for patients and controls separately in a post hoc analysis. To determine if significant correlations between cognitive outcomes and graph measures were more prevalent in hubs than in nonhubs, the McNemar’s test was employed. The significance threshold for all statistical tests was set at *α* < 0.050. We applied Bonferroni correction for multiple comparisons when (a) examining differences in the four whole-brain graph measures (*α* < 0.050/4), (b) correlating six cognitive domain w-scores with significantly different whole-brain (*α* < 0.050/24) and nodal graph measures (see below), and (c) comparing the relative frequencies of significant associations between cognitive scores and four nodal graph measures in hubs versus nonhubs (*α* < 0.050/4). All statistical analyses were conducted using SPSS (Version 28.0.1.1).

## RESULTS

### Participants and Cognitive Data

Out of 397 screened patients, 60 were eligible, and 50 patients participated in the study. Clinical data and demographics for all participants were described previously (De Roeck et al., 2024). In summary, the average age of both groups was 42 years, with an equal distribution of sex (50% male) and educational level (55% with high education per International Standard Classification of Education (ISCED) 2011) among patients and healthy controls. Most patients were diagnosed with WHO Grade 2 oligodendroglioma (54% and 60%, respectively) and had received multimodal treatment: chemotherapy (70%), radiotherapy (76%), and surgery (86%). For glioma survivors, the average time between their last treatment and assessment was 5 years (*SD* = 3,5 years). A heatmap of the lesions is depicted in Supporting Information Figure S1.

Cognitive impairment was found in at least one domain in 60% of glioma survivors, compared with 10% of healthy controls. Across all cognitive domains, patients performed significantly worse (De Roeck et al., 2024).

### Graph Theory Analyses

#### Functional whole-brain graph measures.

Out of four whole-brain graph measures, only whole-brain clustering coefficient values differed significantly between groups, with significantly higher clustering coefficient values (*p*_bonf_ < 0.001, *U* = 462) in patients compared with healthy controls.

#### Functional hub-specific findings.

**Figure 2.**
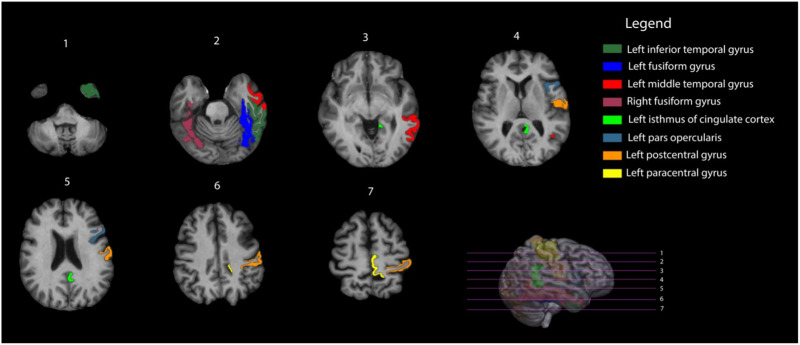
Functional hubs identified in healthy controls (hubscore ≥ 2).

In controls, eight out of 78 nodes were classified as functional network hubs, of which seven were located in the left hemisphere (Figure 2 and Supporting Information Table S2). In patients, the right fusiform gyrus and the left fusiform, middle temporal, paracentral, postcentral gyrus, and pars opercularis were less-frequently defined as a hub compared with controls. While the left inferior temporal gyrus and isthmus of cingulate cortex were more frequently defined as hubs in patients compared with controls, this only reached statistical significance for the left isthmus cingulate (*p*_bonf_ < 0.046).

Forty-one out of 78 nodes were defined as nonhubs in controls, of which 17 were left-sided hubs. In patients, four left-sided nodes (hippocampus, insula, posterior cingulate, and cerebellar cortex) and one right-sided node (caudate) could not be identified as nonhubs, which might indicate network reorganization (Supporting Information Table S3). Floating nodes, defined as nodes with no functional connections to other nodes in the brain, were identified in 31 subjects, with the number of floating nodes per subject ranging from 0 to 8 (Civier et al., 2019).

### Mixed-Design ANOVA for Nodal Graph Measures

Significant differences in clustering coefficients were found between patients and controls, evident across both hub (*F*(1, 98) = 23.679, *p*_bonf_ < 0.001, *η*_*p*_^2^ = 0.195) and nonhub (*F*(1, 98) = 15.093, *p*_bonf_ < 0.001, *η*_*p*_^2^ = 0.133) nodes, with patients consistently exhibiting higher clustering coefficients. Moreover, participant groups exhibited significant differences in hubs for betweenness centrality (*F*(1, 98) = 7.184, *p*_bonf_ = 0.009, *η*_*p*_^2^ = 0.068) and assortativity (*F*(1, 98) = 4.382, *p*_bonf_ = 0.039, *η*_*p*_^2^ = 0.043), with lower centrality but higher assortativity in patients compared with controls. Large effect sizes were observed for the clustering coefficient in hubs (*η*_*p*_^2^ > 0.140), while moderate effect sizes were noted for both betweenness centrality in hubs and clustering coefficient values in nonhubs (*η*_*p*_^2^ between 0.060 and 0.140). Small effect sizes were observed for assortativity in hubs (*η*_*p*_^2^ < 0.060). No differences were encountered in nonhubs for either centrality (*F*(1, 98) = 3.166, *p*_bonf_ = 0.078, *η*_*p*_^2^ = 0.031) or assortativity (*F*(1, 98) = 3.108, *p*_bonf_ = 0.081, *η*_*p*_^2^ = 0.031).

No significant main effect of group was observed for shortest path length, nodal strength, and local efficiency, neither in hubs nor in nonhubs (Table 1). The results of the within-subjects effects are depicted in Supporting Information Table S4, together with a summary of the nodal measures in Supporting Information Table S5. As previous research showed graph measures to change across the life span (Biswal et al., 2010), the analysis was repeated once more with age as a covariate, showing robust results (Supporting Information Table S6).

**Table 1.** Between-subjects mixed-design ANOVAs for hubs and nonhubs

Nodal graph measure	Node group	*df*	*df* (error)	*F* value	*p*_bonf_	*η*_*p*_^2^
Local efficiency	Hubs	1	98	1.685	0.197	0.017
	Nonhubs	1	98	0.327	0.569	0.003
Assortativity	Hubs	1	98	4.382	0.039*	0.043
	Nonhubs	1	98	3.108	0.081	0.031
Clustering coefficient	Hubs	1	98	23.679	<0.001*	0.195
	Nonhubs	1	98	15.093	<0.001*	0.133
Nodal strength	Hubs	1	98	1.267	0.263	0.013
	Nonhubs	1	98	3.553	0.062	0.035
Betweenness centrality	Hubs	1	98	7.184	0.009*	0.068
	Nonhubs	1	98	3.166	0.078	0.031
Shortest path length	Hubs	1	98	1.113	0.294	0.011
	Nonhubs	1	98	0.016	0.899	0.000

### Associations Between Cognitive Domain Scores and Graph Measures

#### Whole-brain graph measures.

Whole-brain graph measures of clustering coefficient correlated negatively with proxy IQ (*r*(98) = −0.409, *p*_bonf_ < 0.001) and positively with executive functioning (*r*(98) = 0.300, *p*_bonf_ = 0.014) in the whole sample. No significant correlations could be found for other cognitive domains.

#### Nodal graph measures.

**Figure 3.**
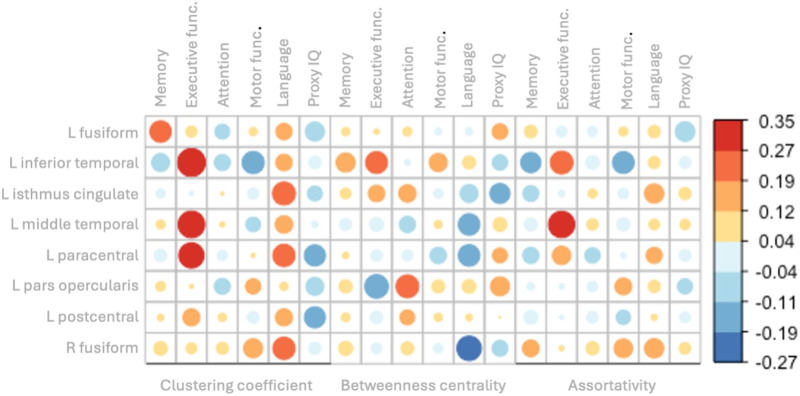
Correlations between graph measures and cognitive domains in hubs. Correlation matrices between cognitive domain w-scores and graph measures of clustering coefficient (left), betweenness centrality (middle), and assortativity (right) in functional hubs. The size of the circle and its color indicate the strength of the association with the highest positive correlation in red and negative correlation in dark blue. L = left, R = right.

**Figure 4.**
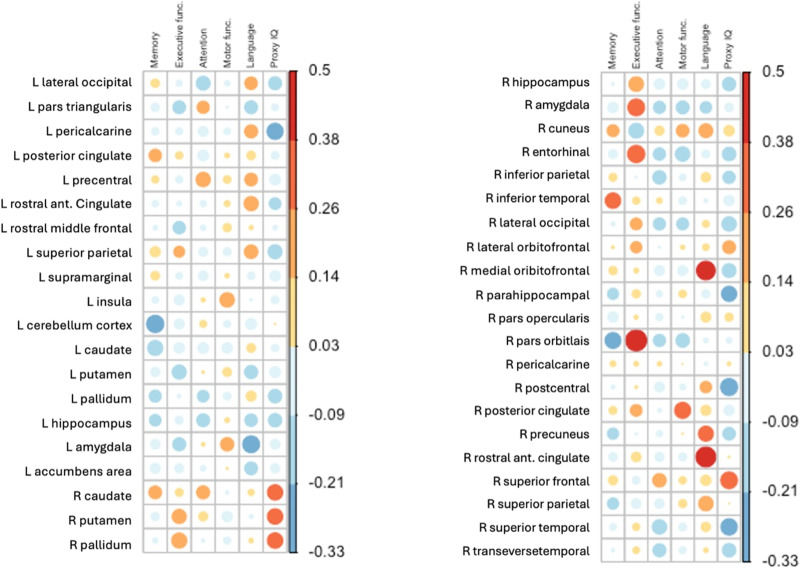
Correlations between clustering coefficient and cognitive domains in nonhubs. Correlation matrices between cognitive domain w-scores and graph measures of clustering coefficient in functional nonhubs. The size of the circle and its color indicate the strength of the association. L = left, R = right.

We analyzed correlations between the six cognitive domain scores and nodal graph measures that significantly differed between groups, that is, betweenness centrality as well as assortativity in hubs and clustering coefficient in both hubs and nonhubs. The results, including significant correlations after Bonferroni correction (*α* < 0.050/882) in the whole sample, are detailed in Figures 3 and 4 and Supporting Information Table S7. Here, we focus on the specific hub and nonhub regions involved, as their relationship with distinct cognitive domains holds clinical significance.

Language outcomes were correlated positively with clustering coefficient values of both the right medial orbitofrontal and rostral anterior cingulate cortex (nonhubs; *r*(98) ≥ 0.396, *p*_bonf_ ≤ 0.009) and negatively with betweenness centrality of the right fusiform gyrus (hub; *r*(98) = −0.300, *p*_bonf_ = 0.049).

Executive functioning was associated with the assortativity value of the left middle temporal gyrus (hub; *r*(98) = 0.308, *p*_bonf_ = 0.049). Moreover, proxy IQ was negatively correlated with the clustering coefficient values of the right postcentral gyrus (nonhub; *r*(98) = −0.329, *p*_bonf_ = 0.044).

Additionally, executive functioning domain scores were also positively correlated with clustering coefficient values of two hub areas (25% of hubs; *r*(98) ≥ 0.317, *p*_bonf_ ≤ 0.030) and two nonhub areas (5% of nonhubs; *r*(98) ≥ 0.337, *p*_bonf_ ≤ 0.040). However, the significance of hubness was not supported (*p* = 0.143), indicating that the clustering coefficient of nodes did not show a significantly higher correlation with cognitive outcomes in hubs compared with nonhubs. However, executive function and language outcomes were associated more consistently with clustering coefficient values in hubs compared with nonhubs.

In a post hoc analysis, we repeated the analysis once more with frontal location as a covariate showing identical results and inpatients and controls separately, showing stronger positive associations between cognitive outcomes and clustering coefficient values in patients compared with controls. Interestingly, in patients and especially in hubs, significant associations between cognitive outcomes and graph measures were mostly positive and more consistently correlated with cognitive outcomes, while in controls and nonhubs, associations with cognitive outcomes were less strong and less consistent. The results are displayed in Supporting Information Figures S2 and S3.

## DISCUSSION

The present study explores the functional brain network topology in glioma patients treated with multimodal therapy and matched healthy controls and its association with cognitive performance. We found the functional brain network in glioma survivors to exhibit greater segregation compared with healthy controls, as evidenced by higher whole-brain and nodal clustering coefficients. These clustering coefficient values were significantly correlated with cognitive functions, particularly executive functioning. In specific hubs, assortativity and betweenness centrality were also associated with executive functioning and language outcomes, respectively. Moreover, hub topology was altered in patients, with the left isthmus cingulate more frequently identified as a hub, potentially indicating reorganization of the functional core network.

Despite the significant variability across functional network studies in brain tumor patients, one of the most consistent findings is the higher segregative properties of these patients’ networks (De Baene et al., 2017; Hart et al., 2016; Liu et al., 2019; Park et al., 2016; van Dellen et al., 2012; Wang et al., 2010; Xu et al., 2013). Our study results align with these findings, evidenced by higher whole-brain and nodal clustering in the network of glioma survivors.

Higher whole-brain clustering in glioma patients indicate that the network nodes across the brain are more densely interconnected with each other, which is assumed to result in improved network efficiency (Watts & Strogatz, 2006). Cognitive functions of general intelligence and executive functioning have been linked to greater network efficiency, since these cognitive functions demand higher capacity for parallel and distributed processing (Li et al., 2009; Na et al., 2018; van den Heuvel et al., 2009; Xu et al., 2013).

Although we found a positive association between executive functioning and clustering coefficient values, patients demonstrated higher clustering coefficients but performed worse on executive functioning tasks, as compared with controls. This suggests that while the brain employs local strategies to preserve cognitive functioning by strengthening local connections, these compensatory mechanisms may not fully offset the disruptive effects of the tumor or its treatment on the network (Stam, 2014), which may result in cognitive (e.g., executive) dysfunction in a significant proportion of patients. Another hypothesis is that this hyperconnectivity results from the recruitment of latent resources to facilitate cognitive task completion, although with reduced efficiency and increased susceptibility to fatigue, ultimately leading to cognitive complaints (Hillary et al., 2015). However, the positive association between whole brain local clustering coefficient values with executive function and the negative association with proxy IQ suggests that, while this compensatory network adaptation is an attempt to preserve cognitive functions such as executive functioning, this network reorganization is detrimental for other cognitive outcomes such as general intelligence.

At the nodal level, patients demonstrated higher local clustering coefficients across both hub and nonhub nodes, indicating an increase in local clustering. Interestingly, this local compensatory mechanism was positively correlated with executive functioning in two left-sided hubs and two right-sided nonhub nodes. However, in post hoc subgroup analyses, we found that higher clustering coefficient values were associated differently across subgroups, showing better cognitive outcomes in patients for both hubs and nonhubs, while higher clustering coefficient values were associated with worse cognitive outcomes for nonhubs in healthy controls. This suggests that the increase in local clustering may serve as a compensatory mechanism to support cognitive functions such as executive functioning in patients, albeit such mechanisms still do not seem to lead to a normative performance.

Furthermore, the distinct associations between cognitive outcomes and clustering coefficient values in patients and controls suggest differing network dynamics. In patients, higher local clustering facilitates cognitive processes by strengthening local networks to compensate for disrupted global connectivity. Conversely, in healthy controls, excessive local clustering in nonhubs might interfere with optimal global network function, leading to poorer cognitive outcomes. These findings imply that what might be an adaptive change in the context of a brain tumor can be maladaptive in a healthy brain or beyond a certain threshold, where balanced global and local connectivity is crucial for optimal cognitive performance.

In addition to potential compensatory mechanisms, maintained cognitive functioning could stem from inherent differences between patients prior to tumor onset, such as variations in preexisting functional network characteristics. Studies indicate that tumors frequently develop in brain regions with specific functional connectivity profiles (Mandal et al., 2020), and the functional connectivity patterns in tumor-affected regions of glioma patients often differ from those typically observed in the same regions of healthy individuals (Derks et al., 2021). This underscores the challenge of directly comparing patients and controls on a one-to-one basis.

Besides the higher clustering of the network nodes in patients, significant differences were also found in betweenness centrality and assortativity in hubs, with glioma patients displaying lower centrality but higher assortativity compared with controls. These group differences suggest that hubs have become less central between other nodes and subnetworks, while they do easily connect to each other, respectively. Regarding behavioral outcomes, betweenness centrality correlated negatively with language outcomes, while assortativity was positively associated with executive functioning in specific hubs. Again, these network changes might act as a compensatory mechanism aiming at preserving higher order cognitive functions such as executive functioning and language. However, they are not sufficient to fully compensate for the network disruptions caused by the tumor or its treatment, ultimately still resulting in cognitive impairment at group level.

Additionally, no significant differences were observed in the shortest path length (integration), nodal strength, and local efficiency, either in hubs or nonhubs. The stability in these network measures suggests that, over the long term, the brain’s functional network adapts to maintain a sustainable, low-cost network. It is also possible that more subtle differences in these network measures exist, which did not reach statistical significance in our sample.

Apart from different functional network properties in patients versus controls, the topology of its hubs did also differ between groups. The increased prominence of the left isthmus cingulate as a hub in glioma survivors may reflect an adaptive process where the brain attempts to maintain functional connectivity by reallocating the hub status to different regions. The left isthmus cingulate is part of the default mode network that is involved in cognitive control and emotional regulation (Roig-Herrero et al., 2022). Its emergence as a hub could indicate a strategic shift to support these critical functions in the face of network disruption in glioma patients. This observation highlights the need for further investigation into the potential vulnerability of this node within the network.

Although associations between clustering and cognitive outcomes were not significantly different between hubs and nonhubs, we did find a more consistent association between clustering and executive function and language outcomes in functional hubs compared with nonhubs, suggesting that clustering of these highly central nodes more consistently seems to impact language and executive functioning more than clustering of nonhubs. This is in line with our previous findings of structural hubs, where assortativity of hubs was significantly more often associated with attention and intelligence outcomes compared with nonhubs (De Roeck et al., 2024). Although there are conceptual reasons to doubt the direct comparability of functional and structural network metrics, our findings suggest that the physical (i.e., white matter tracts) and functional architecture of the brain are impacted differently over time. As proposed in the network failure theory, as a response to a brain lesion, network traffic shifts to highly connected hubs, causing hub overload characterized by heightened activity, metabolism, and functional connectivity. Such prolonged overload of functional hubs may ultimately lead to structural damage, also impacting structural network organization and cognitive functioning (Stam, 2024). This initial phase of increased hub activity and connectivity followed by potential structural deterioration highlights the need for longitudinal studies with early assessments to better understand these temporal dynamics.

### Limitations

The study findings should be interpreted in light of several limitations. Firstly, the choice of atlas may have influenced our results. We opted for the DKT atlas to enable comparison between functional and structural networks in our patient cohort. Nevertheless, many studies in the field utilize functional atlases that may be more sensitive to detecting alterations in functional networks. Future research could investigate the impact of atlas selection on damaged brain networks, as explored in our study.

Secondly, while multiband fMRI allows for faster data acquisition by using multiple slices simultaneously, this acceleration can also lead to signal distortions, particularly in patients with brain tumors. Additionally, it is important to acknowledge that glioma patients in our study predominantly presented with frontal and left-sided lesions, which may introduce a potential selection bias.

Moreover, although we thoroughly checked for extensive signal loss in the frontal and temporal brain regions, we cannot completely rule out the possibility that BOLD signal alterations in these areas may have influenced our results. This limitation is inherent to the patient population, as tumors are often located in these regions.

Furthermore, we did not analyze associations between nodal graph measures that did not differ between groups in either hubs (shortest path length, nodal strength, and local efficiency) or nonhubs (all except for clustering coefficient) and cognitive outcomes.

The lack of preoperative cognitive assessment and imaging limits our ability to differentiate between tumor- and treatment-related factors and their respective impacts on cognition and functional network topology. Moreover, our mixed-design ANOVA model did not include clinical factors. Although a post hoc analysis controlling for age yielded robust results, it is important to consider other factors such as tumor location and volume, grade, IDH1 mutation, edema, and the use of antiepileptic drugs (Kesler et al., 2017; Kirkman et al., 2022; Tariq et al., 2023), which may influence cognitive outcomes. Larger samples and longitudinal measurements are required to explore the potential interactions between these patient-specific features and the functional brain network.

### Strengths

Graph theory analyses can be greatly influenced by methodological choices such as normalization procedures, edge definition and weighting, parcellation schemes, and the criteria for defining hubs and nonhubs. In this study, we employed state-of-the-art imaging sequences and based our methodological choices on the highest reproducibility standards (Ran et al., 2020). Consequently, we constructed weighted networks, using the absolute value of the partial correlation as the weight. We chose partial correlations over full correlations as they take the influence of other regions into account, providing an estimate of relative functional connectivity across the rest of the network (Marrelec et al., 2006; Smith et al., 2011), and the absolute value of these correlations can be interpreted as the amount of information shared between two regions.

Other key strengths of our study include an extensive multimodal assessment and a large sample size within a relatively rare population. Additionally, the inclusion of a control group matched for sex, education, and age allowed for meaningful comparisons between groups. This enabled us to determine whether functional network alterations were present in the survivor group and whether these differences accounted for the observed disparities in cognitive performance between the groups.

### Future Perspectives

Subsequent investigations should delve deeper into the evolving dynamics of network architecture over time, especially concerning various disease stages and treatment modalities. Longitudinal studies will be crucial in uncovering the causal links between network alterations and cognitive deterioration or improvement. While it may be premature to advocate for the routine inclusion of resting-state functional and diffusion scans in clinical practice, the utilization of these advanced imaging sequences to model network changes should be further explored in future research endeavors. By doing so, we can advance our comprehension of cognitive symptoms in this patient population and identify potential targets for behavioral therapies aimed at ameliorating these complaints.

## CONCLUSION

In summary, this study provides compelling evidence of increased network segregation and altered hub topology in glioma survivors compared with healthy controls. These findings might contribute to our understanding of the neural underpinnings of cognitive deficits in this population and underscore the importance of network-based approaches in future research endeavors to understand and address cognitive deficits in this population.

## SUPPORTING INFORMATION

Supporting information for this article is available at https://doi.org/10.1162/netn_a_00449.

## AUTHOR CONTRIBUTIONS

Laurien De Roeck: Conceptualization; Data curation; Formal analysis; Investigation; Methodology; Project administration; Visualization; Writing – original draft; Writing – review & editing. Rob Colaes: Formal analysis; Methodology; Writing – review & editing. Patrick Dupont: Conceptualization; Methodology; Supervision; Writing – review & editing. Stefan Sunaert: Conceptualization; Formal analysis; Methodology; Supervision; Writing – review & editing. Steven De Vleeschouwer: Writing – review & editing. Paul Clement: Writing – review & editing. Charlotte Sleurs: Conceptualization; Formal analysis; Methodology; Supervision; Writing – review & editing. Maarten Lambrecht: Conceptualization; Investigation; Methodology; Supervision; Writing – review & editing.

## FUNDING INFORMATION

Laurien De Roeck, Fonds Wetenschappelijk Onderzoek (https://dx.doi.org/10.13039/501100003130), Award ID: SB/1SE5722N.

## DATA AVAILABILITY

The data that support the findings of this study are available from the corresponding author upon reasonable request. The code is available on the Open Science Framework (link).

## Supplementary Material



## TECHNICAL TERMS

Resting-state functional MRI: A neuroimaging technique that measures spontaneous brain activity by detecting fluctuations in blood oxygenation levels while the subject is at rest (not performing a specific task). It is used to study functional connectivity between different brain regions by analyzing correlated activity patterns (functional connectome).
Average path length: Measures the mean shortest path between all pairs of nodes in a network, providing insights into the overall efficiency of information propagation. The shortest path of a node is the path with minimal cost and the average of the shortest paths from that node to any other node in the network.
Clustering coefficient: Assesses the degree of local connectivity, indicating how well a node’s neighbors are connected to each other. The global clustering coefficient is the average of all nodes’ clustering coefficients.
Hub: A highly connected node within a network that plays a crucial role in facilitating communication between other nodes. In brain networks, hubs act as central regions that integrate and transmit information across different functional or structural areas, supporting efficient neural processing.
Local efficiency: Evaluates the efficiency of information exchange among a node’s immediate neighbors. High local efficiency signifies that the neighbors are well-connected and can efficiently share information.
Global efficiency: Measures the overall interconnectedness of the network.
Nodal strength: Represents the total influence or importance of a node, based on the strength of its connections. Nodes with higher nodal strength are more central and influential within the network due to their strong interactions with other nodes.
Assortativity: Evaluates the tendency of nodes to connect with other nodes that have similar or dissimilar properties.
Betweenness centrality: Measures the frequency of a node appearing on the shortest paths between other nodes, highlighting those crucial for information flow.
Graph theory: A mathematical framework that represents systems as graphs, where nodes signify entities and edges define the relationships between them. It is used to analyze structures and connections in various domains, such as social networks, transportation, and brain connectivity.
