# Supplementary material for: Increased functional network segregation in glioma patients posttherapy: A neurological compensatory response or catastrophe for cognition?
Source: Netw Neurosci. 2025 Jun 27;9(2):743–60. doi: 10.1162/netn_a_00449 (PMC12226146; doi:10.1162/netn_a_00449)

Supplementary Materials

Cognitive data

Raw cognitive assessment scores were transformed into w-scores, which are analogous to z-scores but adjusted for specific covariate(s) to account for the large variability in the sample, i.e. age and in this study. Based on a linear regression model, age- and education specific (ISCED 2011 definition) regression coefficients were calculated based on the healthy controls dataset and obtained test scores were then subtracted from the estimated scores.(1)

As longer response times indicate worse performance, w-scores for time-critical tests were inverted (multiplied by -1) for consistency in interpretation (i.e. higher w-score indicates better performance). Subsequently, these test scores were categorized into six main cognitive domains (Table S1) based on the DSM-V definition of neurocognitive functioning and between-test correlations. (2) The domain summary scores were calculated for all participants by averaging the test-specific w-scores of that particular domain.(3) Cognitive impairment was defined based on the International Cancer and Cognition Task Force recommendations, as two or more test scores with w-scores at or below −1.5 or at least one test score with a w-score at or below −2.0. (4)

References

1. Rijnen SJM, Meskal I, Emons WHM, et al. Evaluation of Normative Data of a Widely Used Computerized Neuropsychological Battery: Applicability and Effects of Sociodemographic Variables in a Dutch Sample. Assessment. 2020;27.

2. Sachdev PS, Blacker D, Blazer DG, et al. Classifying neurocognitive disorders: The DSM-5 approach. Nat Rev Neurol. 2014;10.

3. Andrade C. Z Scores, Standard Scores, and Composite Test Scores Explained. Indian J Psychol Med. 2021;43.

4. Wefel JS, Vardy J, Ahles T, et al. International Cognition and Cancer Task Force recommendations to harmonise studies of cognitive function in patients with cancer. Lancet Oncol. 2011;12.

Supplementary Tables

Table S1 Cognitive (sub)test grouped per cognitive domain

Cognitive domain	Neurocognitive test	Outcome measurement
**Memory**	HVLT-R immediate recall	Sum score - learning
	HVLT-R delayed recall	Sum score
	HVLT-R recognition	Good recognition-mistakes
**Executive functioning**	TMT B	time
	SCWT interference	Interference score
	WAIS IV digit span backwards	Total number of series
	WAIS IV sequencing	Total number of series
**Attention / processing speed**	WAIS IV symbol substitution	Sum score
	TMT A	time
	SCWT colors	time
	SCWT words	time
	WAIS IV digit span forward	Total number of series
**Motor function**	Grooved pegboard	Time (non)dominant hand
**Language**	COWAT semantic	Sum of words
	COWAT phonemic	Sum of words
**Proxy IQ**	WAIS IV matrix reasoning	Sum score

HVLT-R: Hopkins Verbal Learning Test Revised; TMT: Trail Making Test; SCWT: Stroop Color Word Test; WAIS IV: Wechsler Adult Intelligence Scale, fourth edition; COWAT: Controlled Oral Word Association Test.

Table S2 Hub regions and likelihood of being a hub across groups

Hubs	Patients (%)	Controls (%)	two-sided *p*
Left fusiform gyrus	68	74	.509
Left inferior temporal gyrus	70	64	.826
Left isthmus of cingulate cortex	88	72	.046*
Left middle temporal gyrus	74	76	.817
Left paracentral gyrus	58	74	.091
Left pars opercularis	82	84	.790
Left postcentral gyrus	74	82	.334
Right fusiform gyrus	62	74	.198

*: significant value, with threshold of $p<.05$

Table S3 Overview of non-hubs and frequency of designation as a hub in both groups

Non-hub		Patients	Controls
Left	Lateral occipital gyrus	90	84
	Pars triangularis	88	96
	Pericalcarine	98	96
	**Posterior cingulate**	78	88
	Precentral gyrus	96	98
	Rostral anterior cingulate gyrus	98	98
	Rostral middle frontal gyrus	96	88
	Superior parietal gyrus	92	94
	Supramarginal gyrus	92	92
	**Insula**	80	82
	**Cerebellar cortex**	72	92
	Caudate	88	90
	Putamen	100	94
	Pallidum	96	92
	**Hippocampus**	72	82
	Amygdala	96	90
	Accumbens area	100	100
Right	**Caudate**	78	90
	Putamen	86	96
	Pallidum	94	90
	hippocampus	86	82
	Amygdala	88	98
	Cuneus	88	92
	Entorhinal cortex	98	98
	Inferior parietal gyrus	84	84
	Inferior temporal gyrus	100	98
	Lateral occipital gyrus	92	88
	Lateral orbitofrontal gyrus	98	90
	Medial orbitofrontal gyrus	96	98
	parahippocampal	86	84
	Pars opercularis	82	88
	Pars orbitalis	86	88
	pericalcarine	84	86
	Postcentral gyrus	92	86
	Posterior cingulate gyrus	98	98
	Precuneus	98	98
	Rostral anterior cingulate gyrus	98	100
	Superior frontal gyrus	90	94
	Superior parietal gyrus	92	82
	Superior temporal gyrus	98	90
	transverse temporal	98	90

Non-hub (hub score of 0 in>80% of participants of the defined group) in patients compared to controls indicated in grey. Nodes which are defined as non-hub in controls but not in patients are indicated in bold.

Table S4 Within-subjects repeated measures ANOVA

Nodal graph measure	***Node group***	***df***	***df (error)***	***F-value***	***p-value[a]***	***ηp²***
Local efficiency	Hubs	5.522	541.155	1.198	.307	.012
	Non-hubs	10.383	1017.517	4.506	.<.001	.044
Assortativity	Hubs	6.306	617.945	1.721	.110	.017
	Non-hubs	16.307	1598.102	19.313	<.001	.165
Clustering coefficient	Hubs	5.185	508.101	4.782	.<.001	.047
	Non-hubs	14.456	1416.662	8.599	<.001	.081
Nodal strength	Hubs	7	686	2.659	.010	.026
	Non-hubs	22.345	2189.849	16.149	<.001	.141
Betweenness centrality	Hubs	5.882	576.441	3.122	.005	.031
	Non-hubs	15.426	1511.784	6.690	<.001	.064
Shortest path length	Hubs	7	686	4.649	<.001	.045
	Non-hubs	19.824	1942.758	15.592	<.001	.137

Table S5: mean and SD of all graph measures per subject and node group

	Subjects	Node group	Mean	SD
**Local efficiency**	Patients	Hubs	1.034	.037
		Non-hubs	.529	.019
	Controls	Hubs	1.102	.037
		Non-hubs	.514	.019
**Assortativity**	Patients	Hubs	1.736	.092
		Non-hubs	-1.889	.064
	Controls	Hubs	1.463	.092
		Non-hubs	-1.729	.064
**Clustering coefficient**	Patients	Hubs	.938	.008
		Non-hubs	1.203	.007
	Controls	Hubs	.884	.008
		Non-hubs	1.164	.007
**Nodal strength**	Patients	Hubs	1.525	.028
		Non-hubs	.685	.009
	Controls	Hubs	1.570	.028
		Non-hubs	.709	.009
**Betweenness centrality**	Patients	Hubs	3.338	.155
		Non-hubs	.381	.025
	Controls	Hubs	3.926	.155
		Non-hubs	.317	.025
**Shortest path length**	Patients	Hubs	1.161	.024
		Non-hubs	1.815	.034
	Controls	Hubs	1.125	.024
		Non-hubs	1.822	.034

Table S6 Between-subjects repeated measures ANOVA corrected for age (covariate)

Nodal graph measure	*Node group*	*df*	*df (error)*	*F-value*	p_{bonf}	ηp^2
Local efficiency	Hubs	1	98	1.669	.200	.017
	Non-hubs	1	98	.329	.568	.003
Assortativity	Hubs	1	98	4.350	.040*	.043
	Non-hubs	1	98	3.072	.083	.031
Clustering coefficient	Hubs	1	98	24.285	<.001*	.200
	Non-hubs	1	98	14.939	<.001*	.133
Nodal strength	Hubs	1	98	.970	.327	.010
	Non-hubs	1	98	3.661	.059	.036
Betweenness centrality	Hubs	1	98	7.247	.008*	.070
	Non-hubs	1	98	3.392	.069	.034
Shortest path length	Hubs	1	98	1.139	.288	.013
	Non-hubs	1	98	.016	.900	.000

Table S7: Pearson correlations between graph measures and cognitive domain w-scores for hub and non-hub nodes

Graph measure	Node	Group	Memory	Executive func	Attention	Motor func	Language	Proxy IQ
Clustering coefficient	Left inferior temporal gyrus	H		r=.348(p=.030)				
	Left middle temporal gyrus	H		r=.317(p=.004)				
	Right entorhinal cortex	NH		r=.337 (p=.0.04)				
	Right medial orbitofrontal gyrus	NH					r=.396(p=.009)	
	Right pars orbitalis	NH		r=.499(p<.001)				
	Right rostral anterior cingulate cortex	NH					r=.452(p<.001)	
	Right postcentral gyrus	NH						r=-.329(p=.044)
Betweenness centrality	Right fusiform gyrus	H					r=-.300(p=.049)	
Assortativity	Left middle temporal gyrus	H		r=.308(p=.049)				

All p-values are Bonferroni-corrected

Supplementary Figures

Figure S1: Lesion-heatmap of glioma lesions (n=50)

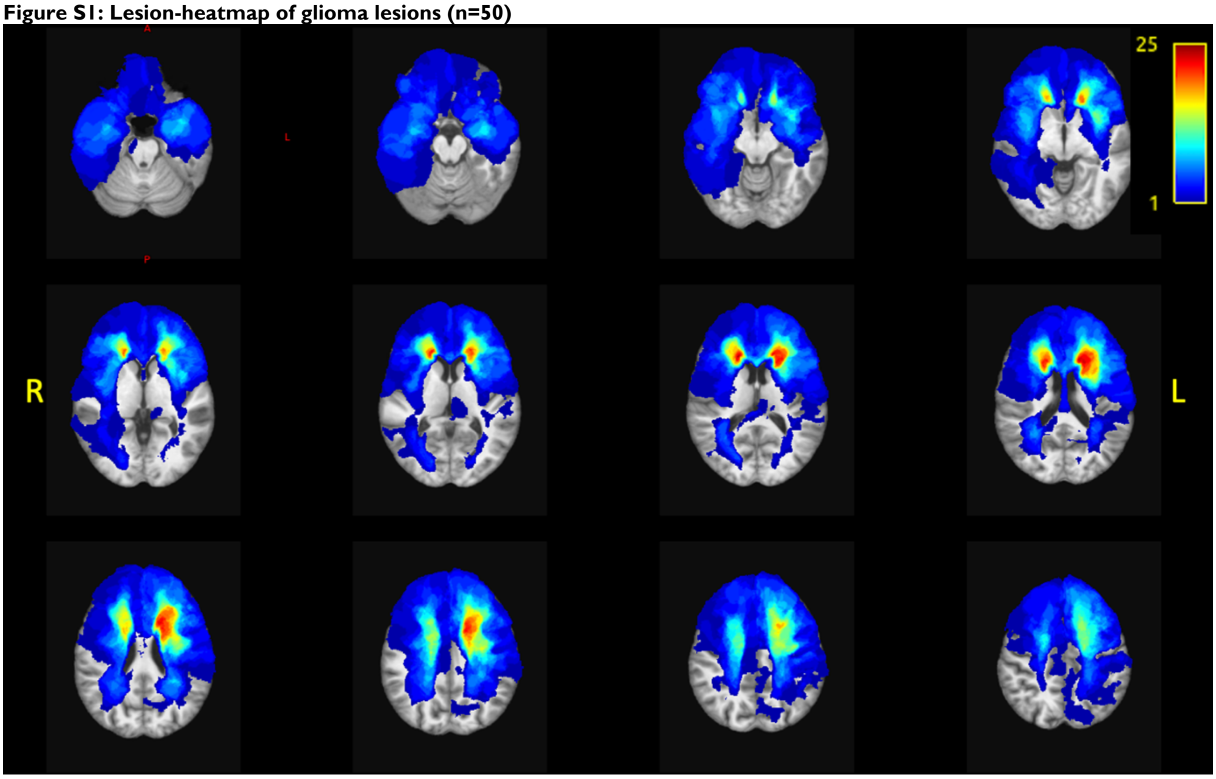

Figure S2: ***Correlations between graph measures and cognitive domains in hubs for patients and controls separately***

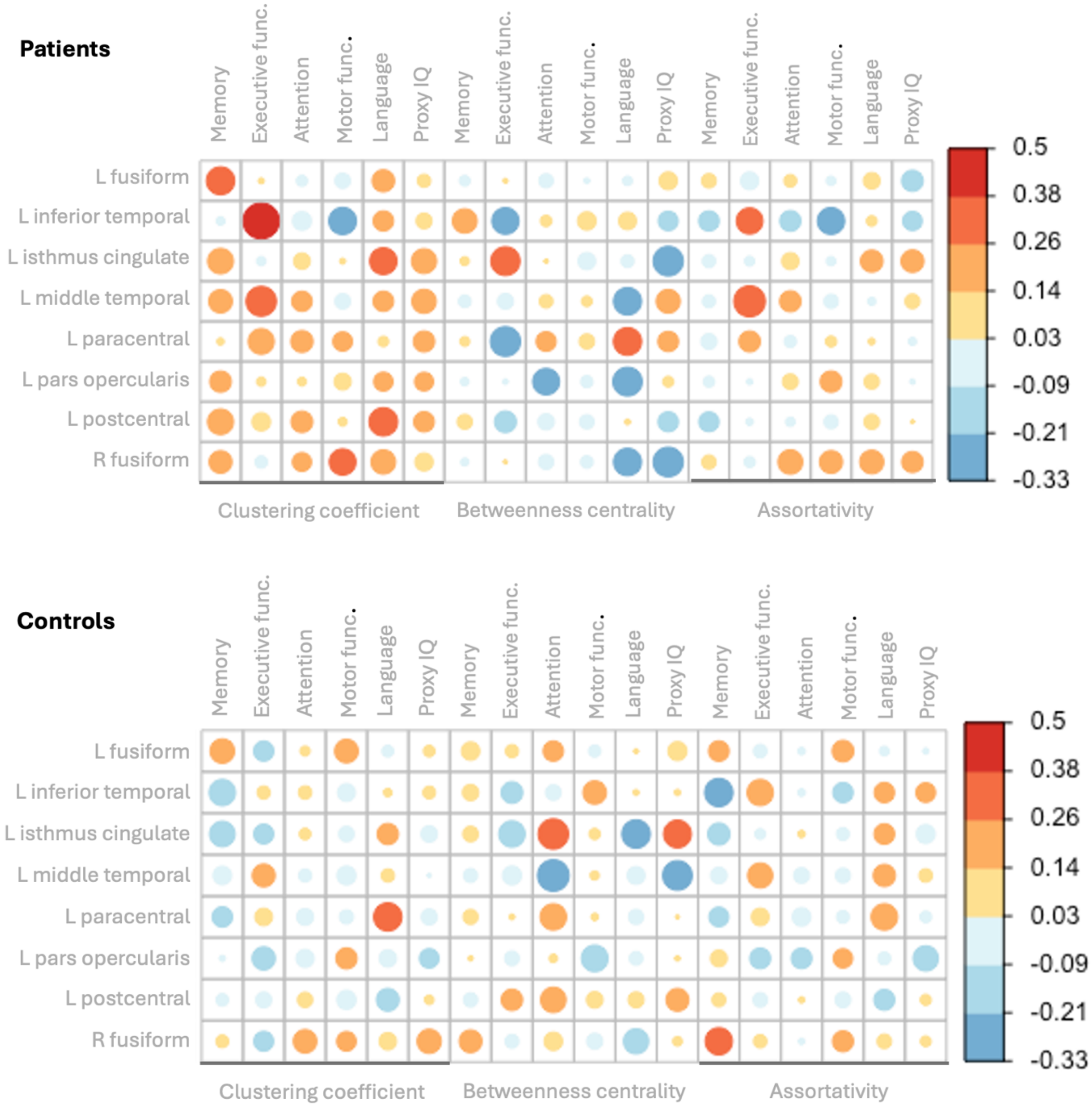

Figure S3: ***Correlations between clustering coefficient and cognitive domains in non-hubs for patients and controls separately***

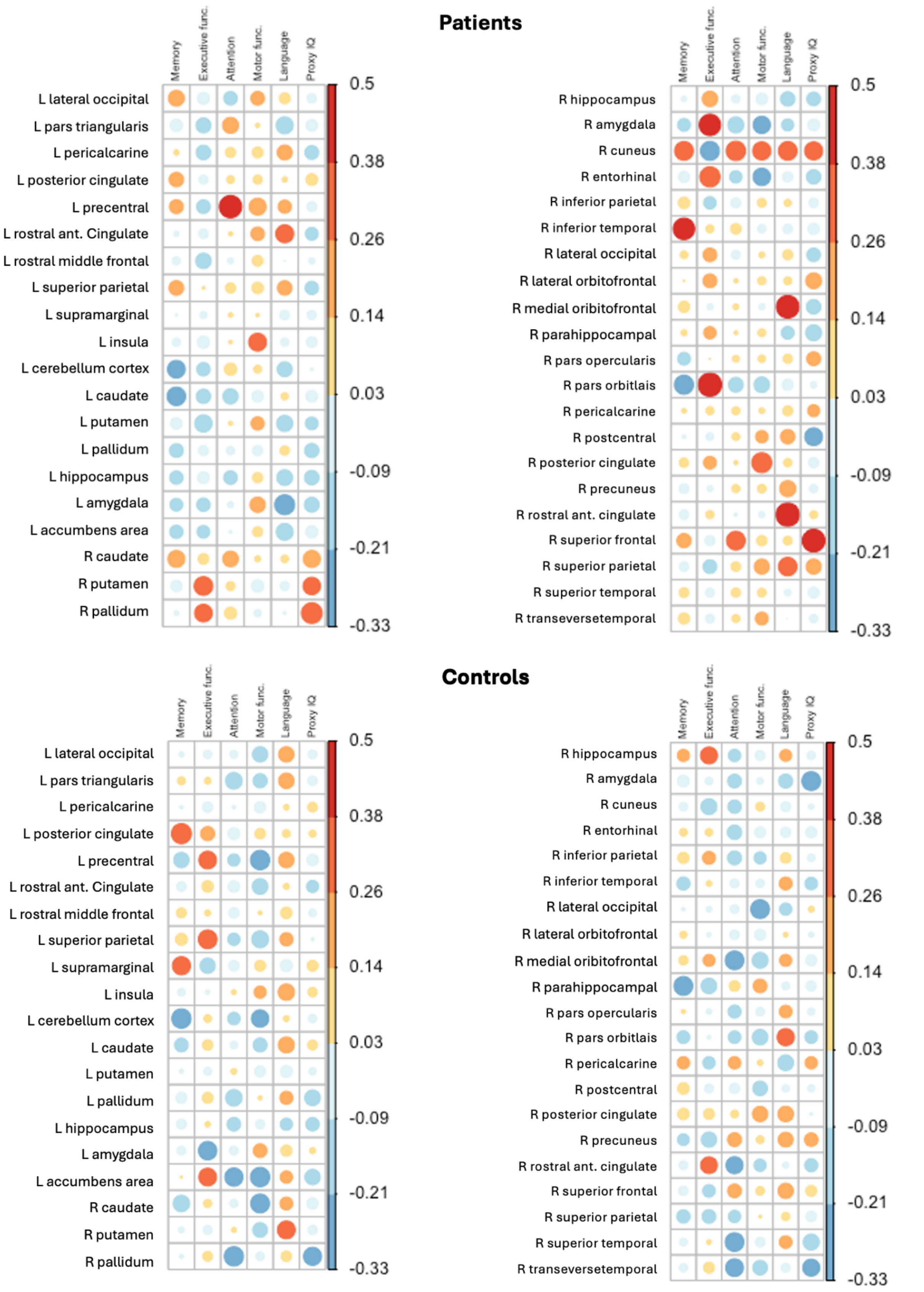

Supplement: Supplementary file 1 [file netn-9-2-743-s001.pdf]
